# Irrigating Solutions and Activation Methods Used in Clinical Endodontics: A Systematic Review

**DOI:** 10.3389/froh.2022.838043

**Published:** 2022-01-31

**Authors:** Riccardo Tonini, Matteo Salvadori, Elisabetta Audino, Salvatore Sauro, Maria Luisa Garo, Stefano Salgarello

**Affiliations:** ^1^Department of Medical and Surgery Specialties, Radiological Sciences and Public Health, Dental School, University of Brescia, Brescia, Italy; ^2^Department of Dentistry, Dental Biomaterials and Minimally Invasive Dentistry, Cardenal Herrera-CEU University, Alfara del Patriarca, Spain; ^3^Department of Therapeutic Dentistry, I.M. Sechenov First Moscow State Medical University, Moscow, Russia

**Keywords:** bacterial load, irrigating solutions, periapical periodontitis, biofilm, root canal agents

## Abstract

**Background:**

*Ex vivo* and *in vitro* studies have demonstrated the effectiveness of some irrigation protocols in reducing the bacterial load in the root canal system. However, standardized protocols have not yet been defined for the real clinical context due to many irrigation procedures available.

**Objective:**

To evaluate the clinical endodontic protocols and limitations of irrigating solutions in the disinfection of the root canal system in patients with apical periodontitis.

**Methods:**

PubMed, Scopus, Embase, Web of Science, and Cochrane databases were searched for randomized controlled trials (RCT) published until January 2021. Hand searching was also performed. Studies focused on evaluating the effectiveness of irrigating solutions and/or irrigation activation methods in reducing the bacterial load in the root canal system were considered. The Cochrane risk-of-bias tool for randomized trials (RoB2) was used to assess the quality of the studies.

**Results:**

Four hundred and twenty eight published articles were identified. After removing the duplicate studies and analyzing full texts, seven RCTs were selected. Two studies compared pure NaOCl with some combination of NaOCl with HEDP and MTAD. Two studies analyzed the antibacterial efficacy of NaOCl and chlorhexidine (CHX). Three studies compared conventional needle irrigation with different irrigation activation methods (PUI, XP-endo finisher, F-file activator, EndoVac activator). The review attained a satisfactory methodology. The main results of each included study were described.

**Discussion:**

Activation methods provide significantly higher biofilm reduction than conventional needle irrigation methods. Combinations of NaOCl with different chelating agents were ineffective in terms of antimicrobial, but it could potentially increase the risk of irrigant extrusion. However, the irrigating protocols were not carefully detailed, especially those regarding the irrigants application time or total volume. The existing literature lacks high-quality studies. The level of evidence is moderate.

**Conclusions:**

The available data is too heterogeneous to compare and identify the superiority of specific valuable irrigation protocols in each clinical context. Application time, volume, and activation methods should be standardized to determine the optimal irrigating procedures to reduce the bacterial load and ensure higher predictability of the endodontic treatment.

**Systematic Review Registration:**

(https://www.crd.york.ac.uk/prospero/display_record.php?RecordID=218555), PROSPERO registration: CRD42020218555.

## Introduction

Apical periodontitis (AP) is a periapical inflammatory response caused by a bacterial infection of the dental pulp [1]. Half of the global adult population has experienced AP in at least one tooth in their lifetimes. The prevalence of AP ranges from 16 to 86% and increases with age [2].

This inflammation is characterized by a complex interplay between microbial tissue invasion and host defense [3]. The defense mechanism keeps the microbial infection in the root canal system, thereby preventing its spread beyond the apical foramen, but the permanence of bacteria in the pulpal tissues leads to pulpal pathology and periapical inflammation [4].

Endodontic therapy aims to remove bacteria, eliminate microbial biofilms and by-products from the root canal system, and prevent subsequent contamination of the intracanal spaces. The reduction of the bacterial load to a level below the one required to assure healing [5] is achieved by combining root canal preparation and disinfection, while the entombment of the low concentration of the surviving bacteria is achieved through proper sealing [6]. The first two steps, i.e., root canal preparation and disinfection, involve enlarging and shaping canals and eliminating bacteria and by-products from even inaccessible and non-instrumented surface areas [7–9]. The second step, i.e., entombment of surviving bacteria, is fundamental for reducing the risk of persistent AP.

To this end, three interplayed strategies are available to achieve substantial bacterial eradication: (1) mechanical instrumentation, (2) irrigation with disinfection solutions, and (3) activated irrigation. Mechanical instrumentation, although necessary to prepare root canals, does not assure their complete disinfection [10]. About 35–53% of the root canal walls remain untouched, biofilm remains *in situ*, smear layers are formed and inaccessible, and non-instrumented surface areas are not disinfected [11–16]. Endodontic disease is, in fact, a biofilm-mediated infection [17] for which the presence of residual biofilms and smear layer reduces the fluid-tight seal of the system [18], does not facilitate root canal disinfection [19, 20], and diminishes filling material adherence, as well as decreasing long-term treatment outcomes [21].

To increase the effectiveness of the root canal disinfection procedures, mechanical debridement is combined with antibacterial irrigants [22]. The chemo-mechanical preparation significantly reduces bacterial load because it acts directly on the root canal walls and allows the antibacterial agents to penetrate the dentinal tubules [16]. Nevertheless, even after chemo-mechanical preparation, microorganisms can remain in the main canal and throughout the root canal system [23–28].

Irrigation activation systems can increase the effectiveness of the irrigations [29]. Activation systems disperse and move the irrigant around the canal system, enhancing chemical surface cleaning and erosion [30], supplementing the antimicrobial effects of chemo-mechanical preparation in infected root canals [26, 27, 31].

Although many *in vitro* and *ex vivo* studies have investigated the antimicrobial efficacy of activated and non-activated irrigants, the irrigant volume, application time, and activation methods have not been uniquely defined to date, and many conflicting or inconsistent results have been reported [32, 33]. Therefore, an update of the review and a further quality assessment of the current literature are required to have an overview of the disinfection procedures currently used in the clinical context. Furthermore, it is necessary to ensure an optimal level of disinfection with high margins of predictability in the endodontic treatment.

This systematic review aimed at evaluating the effectiveness of irrigation procedures in clinical contexts with multispecies bacterial biofilms to identify standardized protocols that can assure comparability of different studies' findings and determine optimal protocols to increase endodontic treatments success rates. Analyzing the bacterial load reduction in patients subjected to AP treatments with varying irrigation procedures can improve clinicians' knowledge and provide helpful information about the most appropriate endodontic irrigation protocol. Thus, only randomized controlled trials (RCT) related to root canal irrigants and their activation techniques were included.

The research questions were:

1. *What is the antibacterial effectiveness of the current irrigating solutions in the root canal system disinfection*?
2. *What is the antibacterial effectiveness of the current irrigation activation systems in the root canal system disinfection?*

## Materials and Methods

### Protocol and Registration

The materials and methods were based on the PRISMA (Preferred Reporting Items for Systematic Reviews and Meta-Analysis) guidelines [34]. The methodology was registered in the PROSPERO (International Prospective Register of Systematic Review) database under the registration number: CRD42020218555.

### Information Sources and Search Strategy

A systematic search was carried out on PubMed, Embase, Web of Science, Scopus, and Cochrane Library from September 2020 to January 2021 without time and language restrictions.

The components of the PICOS question were as follows: (Patients) patients or teeth with AP; (Intervention) irrigating solutions (NaOCl, EDTA, CHX, MTAD) or irrigation activation systems; (Comparison) different irrigation protocols; (Outcome) antimicrobial efficacy measured through (1) the total number of bacteria before and after irrigation and (2) positive result of bacterial samples after irrigation (Study Design) RCT.

### PubMed

The literature search strategy was based on the following key words: ((root canal therapy[MeSH Terms]) OR apical periodontitis OR (periapical periodontitis/therapy^*^[MeSH Terms]) OR (Dental Pulp Cavity[MeSH]) OR (pulpitis[MeSH])) AND ((sodium hypochlorite[MeSH Terms]) OR naocl OR (chlorhexidine[MeSH Terms]) OR CHX OR edetic acid OR mtad OR hedp OR etidronic acid OR EDTA OR Ethylenediaminetetraacetic acid OR saline OR citric acid) AND (irrigant^*^ OR irrigation OR rinse OR disinfect^*^ OR (root canal irrigants^*^[MeSH Terms]) OR (root canal preparation[MeSH Terms]) OR (therapeutic irrigation[MeSH Terms]) OR ultrasonic^*^ OR (ultrasonic therapy[MeSH Terms]) OR Application time OR Volume OR Percentage OR passive activation OR ultrasonic activation) AND (bacterial load^*^ OR smear layer).

Additionally, hand searches were performed in the International Endodontic Journal (1967 onwards) and the Journal of Endodontics (1975 onwards) to identify articles other than those found in the electronic databases. A further hand search of the citation lists of the included studies was performed. Finally, gray literature was searched using the Open Gray database (www.opengrey.eu) with the same search strategy used for the other databases. The first (title/abstract screening) and second (full-text assessment) steps of the search process were performed by two independent reviewers (RT and MLG), and any disagreement was discussed until a decision was made by consensus.

### Study Selection

The complete list of articles obtained through the systematic search was scrutinized to remove duplicates and select, based on the title, the potentially relevant articles to answer the research question. Subsequently, the abstract screening was performed, as well. Two reviewers independently selected the eligible studies (MS and MLG). From the remaining potentially relevant articles, those that met the inclusion and exclusion criteria were selected through full-text reading. Finally, the reasons for exclusion were recorded. The subsequent article selection was independently done by two authors (RT and MLG). When there was disagreement, a third experienced reviewer (SS) was consulted to achieve a consensus.

### Inclusion/Exclusion Criteria

The inclusion criteria were as follows: (i) studies conducted on patients who required endodontic treatment on permanent teeth with a diagnosis of primary or persistent AP or periapical periodontitis; (ii) studies that compared different irrigating solutions or irrigation activation methods; (iii) studies carried out under the CONSORT statement checklist (explicitly or non-explicitly cited); (iv) studies that measured bacterial reduction using bacterial cultivation and/or molecular microbiological methods. Studies performed on primary teeth and/or had no quantitative measure of bacterial reduction were excluded.

### Data Extraction

Two reviewers (MLG and MS) independently extracted the data from the full texts of the studies that fulfilled the inclusion criteria. Disagreements were resolved through team discussions. The primary outcome analyzed in this review was antimicrobial efficacy measured through (1) the total number of bacteria after irrigation or (2) the positive results of bacterial samples after irrigation.

Data extraction was organized in tables that included the following information:

1) Study characteristics: name of the first author, year, intervention arms, number of patients, randomization, and irrigating solution.

2) Participant characteristics: age, sex, type of tooth, and disease.

3) Types of interventions and comparators: irrigating solutions (e.g., sodium hypochlorite, chlorhexidine, EDTA, MTAD, HEDP, saline, or citric acid), activation method, and the volume, application time, and concentration of irrigant.

4) Primary outcome measures: number of total bacteria (CFU/ml or bacteria cells) and/or number of samples with positive bacterial growth after irrigation.

### Data Synthesis

All the data from the eligible articles were synthesized into a narrative summary. The characteristics of each study, which included protocol, type, irrigant concentration and volume, application time, and irrigation activation method, were reported. It was planned to synthesize a quantitative analysis (meta-analysis), but the methodology was not homogeneous among the included studies.

### Risk of Bias Assessment

The quality of each RCT was independently assessed according to the Cochrane Risk of Bias Tool (RoB2) by two reviewers. Five domains of bias (i.e., randomization process, deviations from intended interventions, missing outcome data, measurement of the outcome, and selection of the reported results) were evaluated and reported. The Cochrane Handbook for Systematic Reviews of Interventions [35] was used as a reference guide during the evaluation. A judgment of “high” indicated a high risk of bias, “low” indicated a low risk of bias, and “some concerns” indicated the presence of bias due to lack of information or uncertainty about the potential for bias. Thus, the studies were categorized as having low or high risk of bias or some concerns. The risk of bias was assessed by two authors independently (EA, MLG, and MS). Any discrepancy in the assessment of RoB2 was discussed to attain a consensus.

## Results

### Study Selection

**Figure 1:**
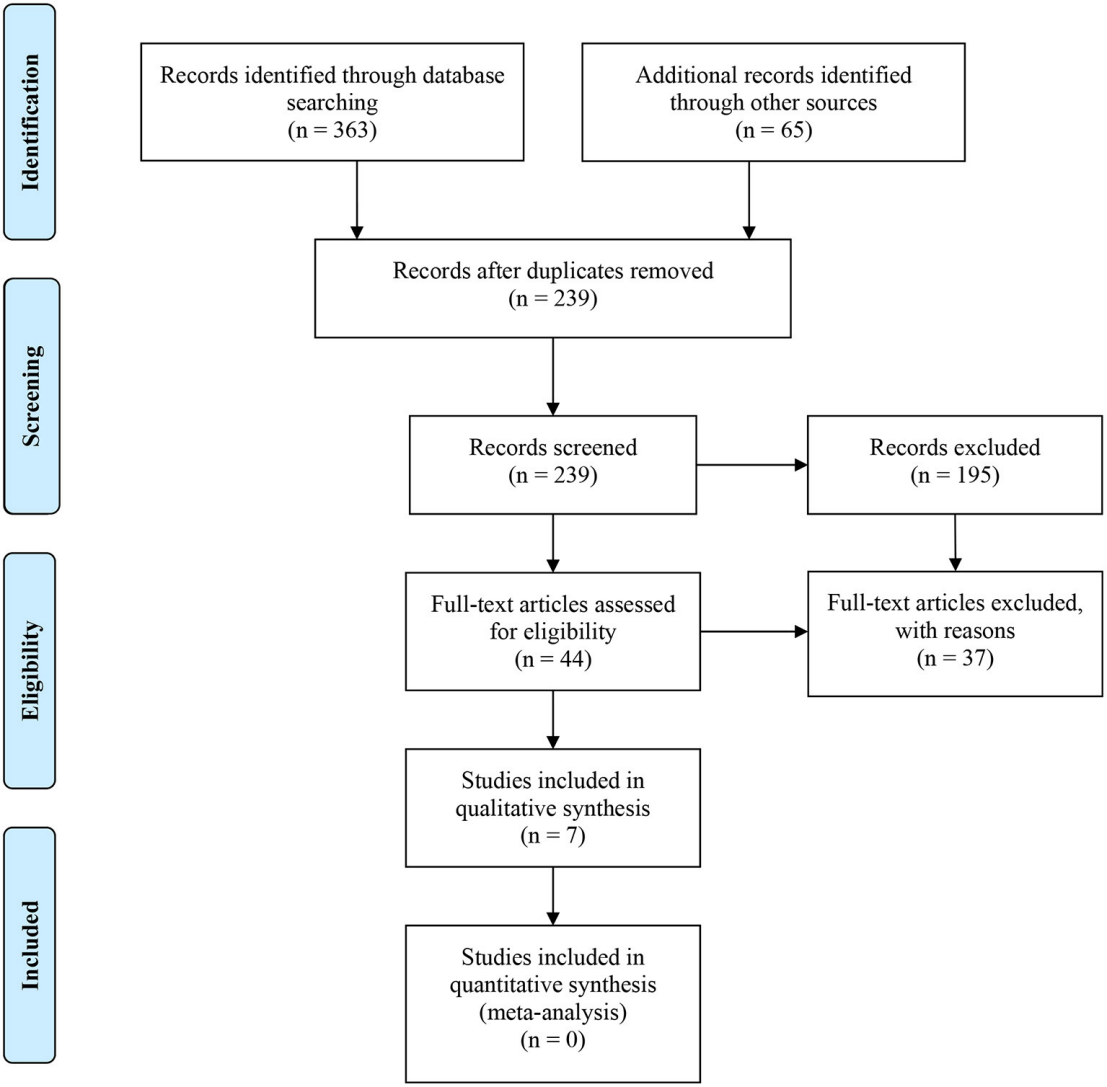
PRISMA 2009 Flow Diagram.

A flow diagram of the search strategy results is presented in Figure 1. After removing 189 duplicates, a total of 239 articles were obtained. From those 239 articles, 195 studies were excluded after reading their titles and abstracts. Finally, 44 studies were selected for full-text reading.

The reasons for exclusion are reported in Table 1. The main reason for the rejection of articles was the lack of RCTs, even though they were classified as *in vivo* or clinical studies. In addition, nine of the rejected papers did not report the bacterial load after the irrigation procedure, eight reported only mechanical instrumentation, two presented non-standardized root canal instrumentation, one analyzed intracanal medication, and one was not available. Therefore, a total of seven studies were included in the systematic review.

**Table 1:** Reasons for exclusion.

Primary Outcome not present	9
No RCT	16
Only mechanical instrumentation	8
Intracanal medication	1
Full text not available	1
No-standardized root canal instrumentation	2
Total studies excluded	37

### Study Characteristics

The data collected from the seven included studies are summarized in Table 2. Three studies compared the effectiveness of irrigation activation systems and that of the conventional irrigation system in reducing the bacterial load [37, 41, 42]. Three studies investigated and compared the efficacy of sodium hypochlorite (NaOCl) alone and that of a combination of NaOCl with dual-rinse HEDP (1-hydroxyethane-1, 1-diphosphonate, or etidronate) [40], BioPure MTAD (mixture of doxycycline, citric acid, and a detergent) and/or chlorhexidine (CHX) [38, 39]. Finally, a study compared MTAD and saline irrigation after 1.3% NaOCl [36]. All studies were conducted considering patients with AP or persistent or refractory AP. Three hundred and twenty-three patients with single-rooted or multi-rooted teeth were included. For each study, the authors considered and included only one straight root per patient.

**Table 2:** Characteristics of the studies.

**First author**	**Year**	**Objective**	**Participants**	**Tooth**				
				**Sample size**	**Type**	**Infectious status**	**Working length**	**Main outcomes**
Malkhassian et al. [36]	2009	To assess the antibacterial efficacy of a final rinse with BioPure MTAD and intracanal medication with 2% CHX	30 (15 males, 15 females, mean age 51.9 years, age range 25–78)	30 (MTAD:15; Saline group: 15)	Single-rooted and multi-rooted teeth (only one root for patient was considered)	Apical periodontitis (primary treatment)	2 mm	Cultivable Bacteria (CFUs/mL) •MTAD: BT: 3.52 × 10^5^ ± 5.83 × 10^5^-AT: 6.04 ± 1.13 × 10^1^ •Saline: BT: 5.41 × 10^4^ ± 1.04 × 10^5^-AT: 6.66 ± 1.01 × 10^1^ •Comparison between groups: no statistically significant difference (*p* > 0.05)
Huffaker et al. [37]	2010	To evaluate the ability of a new passive sonic irrigation system (EndoActivator) and compare it with that of standard syringe irrigation	84 patients	84 (EndoActivator: 42; Needle irrigation: 42)	Not Reported	Apical periodontitis (primary treatment)	1 mm	Detectable bacteria •0.5% NaOCl activated with the EndoVac: AT: 25/42 teeth (60%) •0.5% NaOCl without activation: AT: 27/42 teeth (52%) •Comparison between groups: no statistically significant difference (*p* > 0.05)
Rocas et al. [38]	2016	To compare the antibacterial effectiveness of 2.5% NaOCl and 2% CHX	50 patients (27 males, 23 females, mean age 29 years, age range: 13.52)	50 (2.5% NaOCl: 25; 2% CHX: 25)	Single-rooted teeth	Apical periodontitis (primary treatment)	3 mm	Detectable bacteria •2.5% NaOCl: 25/25 (100%) before treatment−11/25 (44%) after treatment •2% CHX: 25/25 (100%) before treatment−10/25 (40%) after treatment •Comparison between groups: no statistically significant difference (*p* > 0.05) •Number of bacterial cells: •2.5% NaOCl: BT: 1.43 × 10^4^; AT: 5.49 × 10^2^ (*p* <0.001)−95.5% reduction •2% CHX: BT: 8.77 × 10^4^; AT: 2.81 × 10^3^ (*p* <0.001); 95.4% reduction •Comparison between groups: no statistically significant difference (*p* > 0.05)
Zandi et al. [39]	2016	To compare the antibacterial effects of 1% NaOCl and 2% CHX	49 (29 males, 20 females, mean age = 50, age range 21–91)	49 (NaOCl: 20; CHX: 29)	Single-rooted and multi-rooted teeth (only one root for patient was considered)	Apical periodontitis (secondary treatment)	1 mm	Detectable bacteria: •1% NaOCl: 7/20 positive •2% CHX: 12/29 positive •No statistically significant difference between groups (*p* > 0.05) •Number of bacterial cells: •1% NaOCl: BT: 7.96 × 10^4^-AT: 2.95 × 10^2^ (*p* <0.01)−99.6% reduction •2% CHX: BT: 5.37 × 10^5^-AT: 1.10 × 10^3^ (*p* <0.01)−99.8% reduction
Ballal et al. [40]	2019	To assess whether dual rinse HEDP alter the clinical efficacy of NaOCl or adds any untoward clinical effects	60 (35 males, 25 females, age range 18–65 years)	60 (HEDP: 30; NaOCl alore: 30)	Single-rooted and multi-rooted teeth (only one root for patient was considered)	Asymptomatic apical periodontitis (primary treatment)	Determined using an electronic apex locator	Detectable bacteria •HEDP: BT: 30/30–AT: 15/30 •2.5% NaOC: BT: 30/30–AT: 12/30 (40%) •Comparison between groups after treatment: no statistically significant difference (*p* > 0.05)
Ballal et al. [41]	2020	To compare four NaOCl irrigation activation systems	80 (50 males, 30 females, mean age 41)	80 (PUI: 20; F-file: 20; XP-endo finisher: 20; Needle irrigation: 20)	Single-rooted and multi-rooted teeth (only one root for patient was considered)	Asymptomatic apical periodontitis with and without periapical lesions	Determined using radiographs and an apex locator	Cultivable Bacteria (CFUs/mL) •XP-endo Finisher: BT: median: 12.20; sd: 45.87–AT: median: 0.008; sd: 0.0001 •Needle irrigation: BT: median: 12.40; sd: 9.2–AT: median: 1.09, sd: 3.56 •F-files: BT: median: 20.65, sd: 69.23–AT: median: 0.34, sd: 4.72 •Ultrasonic: BT: median: 44.82, sd: 16.60–AT: median: 0.0055; sd: 0.032
Orozco et al. [42]	2020	To evaluate the effectiveness of passive ultrasonic irrigation compared to conventional needle irrigation	20 (10 females, 10 males)	20 (PUI: 10; Needle irrigation: 10)	Single-rooted and multi-rooted teeth (only one root for patient was considered)	Primary endodontic infection	1 mm	Cultivable Bacteria (CFUs/mL) •PUI: BT: 25.8 × 10^5^ ± 4.70 × 10^5^-AT: 42 ± 119 •Needle irrigation: BT: 2.31 × 10^5^ ± 4.70 × 10^5^-AT: 1.76 × 10^3^ ± 3.31 × 10^3^ •Comparison between groups after treatment: no statistically significant difference (*p* > 0.05)

*AT, After Treatment; BT, before treatment; PUI, Passive Ultrasonic Irrigation*.

### Risk of Bias

**Figure 2:**
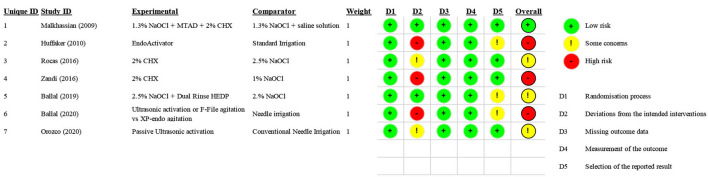
Risk of Bias—ROB2.

One study was considered to have a “low” risk of bias [36], three studies were considered to have “some concern [39, 40, 42], and three studies were considered to have a “high” risk of bias [37, 38, 41] because of a lack of blinding. The risk of bias of each randomized clinical trial is reported in Figure 2.

### Irrigating Solutions

The effectiveness of the irrigating solutions was investigated in four studies, which together included 189 patients with AP (Table 3) [36, 38–40]. Overall, all studies found no differences between NaOCl alone and the combination of NaOCl with HEDP and/or CHX. Malkhassian et al. compared 1.3% NaOCl with MTAD and 2% CHX combined with 1.3% NaOCl and saline solution in a sample of 30 patients [36]. Conventional needle irrigation was used for 5 min in both groups. The total volume of irrigants was 10.5 mL of 1.3% NaOCl and 5 mL of MTAD for the treatment group and 10.5 mL of 1.3% NaOCl and 5 mL of saline solution for the control group. Although the treatment group reported a lower bacterial count than the control group, the final rinse with MTAD and medication with CHX had no significant reduction on the biofilm beyond the level registered using NaOCl and saline solution (*p* > 0.05).

**Table 3:** Studies comparing irrigating solutions.

**References**	**Irrigant (Treatment group vs. Control group)**	**Total volume**	**Application time**	**Irrigation technique**
Malkhassian et al. [36]	1.3% NaOCl + MTAD + 2% CHX vs. 1.3% NaOCl + saline solution	10.5 mL NaOCl + 5 mL MTAD or saline solution	5 min	Needle irrigation
Rocas et al. [38]	2.5% NaOCL vs. 2% CHX	15 mL NaOCL or 1 mL CHX	Not Reported	Needle irrigation
Zandi et al. [39]	1% NaOCl vs. 2% CHX	10 mL	Not Reported	Needle irrigation
Ballal et al. [40]	2.5% NaOCl + 9% HEDP vs. 2.5% NaOCl	25 mL	1 min	Needle irrigation

Rocas et al. [38] compared the effectiveness of 2% CHX with that of 2.5% NaOCl using a total volume of 15 mL for both irrigants but did not report the application time. In both groups, the mean number of bacterial cells decreased significantly after irrigation (*p* < 0.01). The rate of reduction in detectable bacteria was 40 and 44% in the treatment group (2% CHX) and in the control group (2.5% NaOCl), respectively. However, no statistically significant difference was observed upon comparing the mean number of bacterial cells between groups (*p* > 0.05) [38].

Zandi et al. [39] compared the effectiveness of 2% CHX with that of 1% NaOCl using a total volume of 10 mL for both irrigants but did not report the application time. In both groups, the mean number of bacterial cells decreased significantly after irrigation (*p* < 0.01), and the rate of reduction was higher than 99% (99.6% in the treatment group and 99.8% in the control group). However, no statistically significant difference was observed upon comparing the detectable bacteria between groups (*p* > 0.05).

Ballal et al. [40] investigated whether dual-rinse HEDP alters the clinical efficacy of NaOCl or adds any untoward clinical effects in a sample of 60 patients with AP, but the authors pointed out that the aim of their study was not to simulate a clinical scenario. Pure 2.5% NaOCl and 2.5% NaOCl combined with 9% HEDP were compared. With the use of 25 mL in both groups and exposure of 1 min, it was found that the 2.5% NaOCl/dual-rinse HEDP mixture made 15 out of 30 (50%) canals free of microorganisms. In contrast, irrigation with pure 2.5% NaOCl rendered 12 out of 30 (40%) canals free of microorganisms. This numerical difference was not characterized by statistical significance (*p* = 0.60). Microbiological analysis revealed the presence of 6 anaerobic species in the NaOCl group and seven in the NaOCH + HEDP group. After irrigation, no apparent aerobic or anaerobic taxa selection occurred in either group [40].

### Activation Methods

Three studies [37, 41, 42] compared the effectiveness of conventional needle irrigation and that of activation procedures such as passive ultrasonic irrigation (PUI) [41, 42], EndoActivator [37], and F-file agitation, and XP-endo finisher agitation [41] (Table 4).

**Table 4:** Studies comparing activation methods.

**References**	**Irrigant**	**Total volume**	**Application time**	**Activation technique**
Huffaker et al. [37]	0.5% NaOCl	Not Reported	1 min	Endo Activator
				Standard irrigation
				Non-activated single-irrigation
				Non-activated irrigation
Ballal et al. [41]	2.5% NaOCl (5 mL)	25 mL	1 min	Needle irrigation
				Passive ultrasonic activation
				F-File agitation
				XP-Endo Finisher
Orozco et al. [42]	2.5% NaOCl (4 mL) + 17% EDTA	24 mL NaOCl—EDTA not reported	3 min	Passive ultrasonic irrigation
				Convention needle irrigation

In the two studies, PUI obtained significantly better results than needle irrigation did [41, 42] when the NaOCl concentration was set to 2.5% and used alone [41] or in combination with 17% EDTA [42] for 1 min and with different total volumes (25, 40, or 24 mL). Moreover, Orozco et al. [42] showed a higher presence of *S. constellatus, P. gingivalis*, and *A. actinomycetemcomitans* in the PUI group, and *F. nucleatum sp. vicentii, L. buccalis*, and *S. mitis* in the control group, without however registering statistically significant differences (*p* > 0.05).

One study compared EndoActivator and needle irrigation using 0.5% NaOCl alone [37]. After activating 0.5% NaOCl with the EndoVac activator, 25 teeth (60%) still harbored cultivable bacteria. In comparison, 27 teeth (52%) harbored cultivable bacteria in the control group, and no statistical significance emerged (*p* > 0.05) between the two groups [37].

## Discussion

Irrigation plays a crucial role in treating AP because it can reduce the bacterial load to ensure long-term healing. An ideal root canal irrigation process should remove the bacteria, biofilm, and smear layer and disinfect all parts of the root canal system, including anatomical complexities. Irrigating solutions and activation methods should be combined to achieve better cleanliness, reduce the adverse effects of irrigants on the physical properties of exposed dentine, and improve the sealing ability of the filling materials [43].

### Irrigating Solutions

Sodium hypochlorite (NaOCl) is the most commonly used irrigant because of its antimicrobial activity, ability to dissolve organic matter [44], and low cost. Irrigant frequent exchanges and a greater volume are recommended for improving its effectiveness [45, 46]. The disadvantages of NaOCl are its significant toxicity when accidentally injected into the periradicular tissue, disagreeable smell and taste, and risk of bleaching clothes and corroding metal objects [43]. Moreover, NaOCl significantly affects mechanical properties of dentine, such as microhardness, roughness, elastic modulus, flexural strength, inorganic content, and organic-inorganic ratio [47, 48]. Some authors found 1.3% NaOCl and 2.5% NaOCl are ineffective in removing bacterial load [49]. Moreover, NaOCl is ineffective in removing the inorganic components of the smear layer and the hard-tissue debris that accumulates during mechanical instrumentation [50].

Therefore, the combination of NaOCl with MTAD and HEDP could result in an optimal irrigation mixture. The ability of MTAD and HEDP to remove the smear layer and the inorganic components left in the canal during the mechanical instrumentation was tested using MTAD and HEDP in combination with two different NaOCl concentrations (i.e., 2.5 and 1.3%) for 1 and 5 min, respectively [36, 41]. Although both mixtures reduced the bacterial load to a level below that required, no differences emerged upon comparing them with NaOCl alone. Both HEDP and MTDA showed high effectiveness in completely removing the smear layer. For the latter, its action is enhanced when a low concentration of NaOCl (1.3%) is used. For HEDP, several beneficial effects have been reported: (1) prevention of smear layer [51], (2) reduction of hard tissue debris accumulation [52], (3) possible reduction of torsional load on rotary instrumentation [53], (4) time-saving application [40], and (5) not reduction of NaOCl antibacterial effect [54].

Chlorhexidine (CHX) is considered an alternative to NaOCl because of its antibacterial properties [55], effectiveness (which lasts for days or weeks), and capability to prevent root canal reinfection [56, 57]. Contrary to the characteristics of NaOCl, CHX is substantive to dentin [58] and results in less tissue irritation [59], although its effective role in the disruption of polymicrobial biofilms [60] and dissolving pulp tissue remnants [61] is still debated. Moreover, a recent study showed a substantial reduction of CHX effectiveness in the long term because of the electrostatic attraction of CHX to extracellular polymeric substances, limiting CHX penetration and reducing its concentration in deep biofilm layers [62].

In two studies, an increased CHX concentration of 2% reduced the bacterial load as effective as 1% NaOCl [38, 39], but the role of CHX in the reduction of the bacterial load continues to be uncertain, as emerged in Gonçalves et al. [63] and Fedorowicz et al. [64].

Moreover, according to Goncalves et al. [63], our findings showed that the application time of irrigants has continued to be not reported in the included studies, so the potential role of the time in the effectiveness of NaOCl or CHX remains unclear [63].

Overall, *in-vivo studies* comparing irrigating solutions did not report statistically significant differences in reducing the bacterial count, so contradicting results emerged in *in-vitro* studies. This could explain by several factors. First, some studies were underpowered because of a substantial reduction of bacteria density after root canal preparation or a limited initial number of samples [36, 39]: an initial bacterial count higher than 2.7 × 10^4^ cell equivalents could reduce the effectiveness of irrigating solution showing a prevalence of bacterial in 14 out of 19 cases (74%) [39]. Irrigating solutions application time was missing in two studies out of four: this prevented us from understanding a direct relationship between application time and irrigation. In the two studies where application time was reported, the enormous difference in time (1 min vs. 5 min) between them does not indicate a positive association with bacterial reduction. Probably, other variables should be considered in such kinds of clinical studies as present species and their spatial location in the canal system, their access to nutrients, and their ability to adapt to and survive [5].

### Activation Methods

Two studies compared the effectiveness of ultrasonic activation methods in reducing bacterial compared to conventional needle irrigation [40, 42]. In both cases, ultrasonic irrigation showed statistically significant action in reducing the bacterial load. As emerged in many studies, ultrasonic activation methods are fundamental to the effectiveness of irrigants [41, 42]. They are based on the transmission of acoustic energy through the irrigant via a stainless steel wire or endodontic file [65]. Acoustic energy is dissipated through the irrigant, leading to cavitation and microstreaming; this allows the irrigant to move dynamically and thoroughly within the canal system [66, 67]. Acoustic waves produce cavitation bubbles; the energy released after bubble collapse is transmitted to the root canal walls, liberating the debris found [37]. Microstreaming then carries the debris coronally to remove it from the canal [37]. The effective action of PUI has been explained as the result of node production along activated files, and therefore a strong current production along the activated instrument [67]. The presence of several nodes along the instrument prevents reducing acoustic streaming reduction when the file touches the canal wall. However, while microstreaming is a biophysical force strongly associated with endodontic files, the role of cavitation *in vivo* is debatable [68]. The combination of acoustic streaming and cavitation could be considered a critical element in the most effective action of the ultrasonic activation method.

One study comparing the effectiveness of sonic activation (EndoActivator) and conventional needle irrigation [37] in reducing the bacterial load did not report statistically significant differences. However, the two methods are not interchangeable. Although EndoActivator is considered less performant than ultrasonic activation, due to the production of only one node along the length of the instrument, the similar efficacy of irrigants registered in Huffaker et al. [37] through the needle irrigation method is not related to physical action, but it is likely attributable to the irrigant antimicrobial properties. Moreover, conventional needle irrigation may fail to deliver irrigants in the apical third, where entrapped gas particles may produce a vapor lock effect [69], although this effect could be prevented when the root canal is enlarged adequately and the needle is placed close to working length [70]. Besides, conventional needle irrigation generates a positive pressure at the end of the needle forcing the irrigating solutions and microbial debris into the periapical tissue. Combining NaOCl and chelating agents such as HEDP can potentially reduce debris accumulation in the apical parts, but it can force irrigants into periapical tissue [71] if positive pressure is applied. Finally, depending on the needle tip, the extent of irrigation delivery beyond the needle tip may change: for open-ended needles, the jet is intense and extends apically to the needle tip along the root canal, while for closes-ended needles, the jet is formed near the apex of the outlet and it is directed apically with a slight divergence [72]. On the contrary, EndoActivator applies a negative pressure to irrigate and remove debris from the apex without forcing the irrigant into the periapical tissues, so resulting in more effective than conventional needle irrigation in the clinical context because it reduces the risk of overirrigation.

One study compared the effectiveness of PUI with those reported, respectively, using XP-endo finisher, F-File, and needle irrigation [40]. While a statistically significant reduction in CFUs emerged comparing the first three methods and needle irrigation, and PUI or XP-endo with F-file, no statistically significant difference emerged between XP-endo finisher and PUI. These comparable results should be explained as the result of XP-endo finisher capacity to react at various temperature levels, which allows the instrument to modify from its straight shape to a unique spoon shape at body temperature, adapting its shape to that of the root canal in a three-dimensionally manner. The positive action of XP-endo finisher was confirmed in some *ex vivo* and *in vitro* studies [73–76] showing a solid effectiveness action of XP-endo finisher in removing the accumulated hard-tissue debris, smear layer, and microbes from the root canal system.

In the same study, the F-file, a plastic rotary finishing file, also resulted in a more significant reduction of CFUs when compared to needle irrigation. The design of the F-file, characterized by a diamond abrasive embedded into a non-toxic polymer of 20 mm at the tip with a 0.04 taper, removes dentinal wall debris and agitates the irrigant without further enlarging the canal [77]. From our findings, F-file activation reported a significantly lower reduction of CFUs compared to PUI or XP-endo finisher: results on the effective role of F-file appears controversial. This result, in fact, contradicted what emerged in a laboratory study that reported the non-inferiority of F-file when compared with PUI [78]. In addition, the F-file showed greater effectiveness in removing smear layer and debris [79, 80], both *in vivo* and *in-vitro* studies, but its role in the reduction of the whole bacterial load in clinical context appears not as effective as PUI or XP-endo finisher.

Overall, NaOCl and agitation methods (e.g., PUI or XP-endo finisher) promoted a better irrigant distribution in the root canal system. Nevertheless, some concerns remain unresolved. Irrigants such as NaOCl can produce ultrastructural alterations in the dentin collagen and promote peritubular and intratubular erosion, especially if enhanced by PUI [81]. All the included studies analyzed the effectiveness of the irrigation procedures using straight roots. A unique, real clinical irrigation protocol has not been defined yet. Many variables that influence the success of endodontic disinfection are unstandardized and remitted to operators based on the patients' anatomical conditions, as operator experience, apical enlargement, axial pressure, choice of the irrigating or instrumentation sequence [82, 83], or canal morphology [84].

From our findings, the role of ultrasonic activation, although not wholly defined, seems to be fundamental in reducing the bacterial load. This superior role of ultrasonically activated irrigation also emerged in Nagendrababu et al. [85], who compared ultrasonically active irrigations with other irrigation techniques. Nevertheless, the role of ultrasonic activation in improving the healing rate of apical periodontitis compared with syringe irrigation continues to be not well-defined [86], although it has a crucial role in reducing post-operative pain and improving canal and isthmus cleanliness during the endodontic treatments [87].

### Limitations

The main goal of this work was to review simultaneously analyze irrigating solutions and activation methods in a clinical context. Although it was possible to determine the main results of each included study, describing their protocols was more complex, and comparing the results was impossible due to the heterogeneity of the methodologies. In addition, information about the application time and volume of irrigants was not always available. In this review, cytotoxic of irrigants was not treated. Still, it remains an essential topic in the choice of irrigating solutions and should be considered in identifying standardized protocols.

The existing literature lacks high-quality studies, and the level of evidence is moderate. In addition, the lack of double-blind procedures, which are sometimes not easy to implement, and some concerns regarding the selection of the reported results reduced the quality of the studies.

In *in-vivo* studies, sampling from root canals is more complex than *in-vitro* studies, and it still relies mainly on paper points. Samples represent only the condition in the main root canal but do not evaluate microbial load in non-instrumented areas, isthmuses, and lateral canals. This means that the efficacy of irrigants or activation methods is not reflected accurately. This discrepancy between *in-vivo* and *in-vitro* studies combined with a lack of a universal standard for assessing the antibacterial efficacy of endodontic treatment prevents determining standard protocols of optimal irrigation methods.

This systematic review raises questions about the optimal irrigating solutions and their concentrations, volumes, and application times.

### Future Research

RCTs complying with the PRIRATE 2020 guidelines [88, 89] are needed to define standardized measuring protocols and develop comparable irrigation procedures to improve the assessment of the different protocols.

The application time, volume, activation methods, and retention time of irrigants as well as the irrigation flow, needle type, and depth of needle placement are factors that influence the penetration of irrigants, but there is a lack of proper data regarding their ability to improve penetration and filling.

## Conclusions

Considering the limitations of the selected studies and the review itself, it should be acknowledged that activated irrigation procedures are fundamental for reducing the bacterial load in the whole root canal system and that NaOCl continues to be a key element in all protocols.

Improving the quality of studies requires identifying more efficient protocols and more suitable approaches that facilitate the work of clinicians, reduce chairside time, and have more reliable outcomes. Combining potent irrigating mixtures and activation methods can lead to higher outcome predictability and lower risk of side effects. A standardized protocol is desirable.

When developing a new approach, the application time, volume, and activation method should be standardized to uniquely define protocols and improve our knowledge about the actual effectiveness of the irrigating procedures in real clinical contexts. High-quality clinical studies are essential for determining the optimal approach to endodontic treatment and defining the strengths and limitations of each available procedure to assure patients with the highest-possible level of success predictability.

## Data Availability Statement

The original contributions presented in the study are included in the article/supplementary material, further inquiries can be directed to the corresponding author/s.

## Author Contributions

RT and MG: conceptualization. MG and MS: methodology and software. RT, EA, MG, and SSal: validation. MG, RT, and MS: writing-original draft preparation. SSau and SSal: writing-review and editing. MG and RT: supervision. RT: project administration. All authors contributed to the article and approved the submitted version.

## Conflict of Interest

The authors declare that the research was conducted in the absence of any commercial or financial relationships that could be construed as a potential conflict of interest.

## Publisher's Note

All claims expressed in this article are solely those of the authors and do not necessarily represent those of their affiliated organizations, or those of the publisher, the editors and the reviewers. Any product that may be evaluated in this article, or claim that may be made by its manufacturer, is not guaranteed or endorsed by the publisher.
