# Corrigendum: Irrigating Solutions and Activation Methods Used in Clinical Endodontics: A Systematic Review

**DOI:** 10.3389/froh.2022.876265

**Published:** 2022-03-24

**Authors:** Riccardo Tonini, Matteo Salvadori, Elisabetta Audino, Salvatore Sauro, Maria Luisa Garo, Stefano Salgarello

**Affiliations:** ^1^Department of Medical and Surgery Specialties, Radiological Sciences and Public Health, Dental School, University of Brescia, Brescia, Italy; ^2^Department of Dentistry, Dental Biomaterials and Minimally Invasive Dentistry, Cardenal Herrera-CEU University, Alfara del Patriarca, Spain; ^3^Department of Therapeutic Dentistry, I.M. Sechenov First Moscow State Medical University, Moscow, Russia

**Keywords:** bacterial load, irrigating solutions, periapical periodontitis, biofilm, root canal agents

In the original article, there was a mistake in Table 2, as published. In the column “Main Outcome,” there were non-clear indications of outcomes. The corrected Table 2 appears below.

**Table 2:** Characteristics of the studies.

**First author**	**Year**	**Objective**	**Participants**	**Tooth**				
				**Sample size**	**Type**	**Infectious status**	**Working length**	**Main outcomes**
Malkhassian et al. [36]	2009	To assess the antibacterial efficacy of a final rinse with BioPure MTAD and intracanal medication with 2% CHX	30 (15 males, 15 females, mean age 51.9 years, age range 25–78)	30 (MTAD:15; Saline group: 15)	Single-rooted and multi-rooted teeth (only one root for patient was considered)	Apical periodontitis (primary treatment)	2 mm	Cultivable Bacteria (CFUs/mL) •MTAD: BT: 3.52 × 10^5^ ± 5.83 × 10^5^-AT: 6.04 ± 1.13 × 10^1^ •Saline: BT: 5.41 × 10^4^ ± 1.04 × 10^5^-AT: 6.66 ± 1.01 × 10^1^ •Comparison between groups: no statistically significant difference (*p* > 0.05)
Huffaker et al. [37]	2010	To evaluate the ability of a new passive sonic irrigation system (EndoActivator) and compare it with that of standard syringe irrigation	84 patients	84 (EndoActivator: 42; Needle irrigation: 42)	Not Reported	Apical periodontitis (primary treatment)	1 mm	Detectable bacteria •0.5% NaOCl activated with the EndoVac: AT: 25/42 teeth (60%) •0.5% NaOCl without activation: AT: 27/42 teeth (52%) •Comparison between groups: no statistically significant difference (*p* > 0.05)
Rocas et al. [38]	2016	To compare the antibacterial effectiveness of 2.5% NaOCl and 2% CHX	50 patients (27 males, 23 females, mean age 29 years, age range: 13.52)	50 (2.5% NaOCl: 25; 2% CHX: 25)	Single-rooted teeth	Apical periodontitis (primary treatment)	3 mm	Detectable bacteria •2.5% NaOCl: 25/25 (100%) before treatment−11/25 (44%) after treatment •2% CHX: 25/25 (100%) before treatment−10/25 (40%) after treatment •Comparison between groups: no statistically significant difference (*p* > 0.05) •Number of bacterial cells: •2.5% NaOCl: BT: 1.43 × 10^4^; AT: 5.49 × 10^2^ (*p* < 0.001)−95.5% reduction •2% CHX: BT: 8.77 × 10^4^; AT: 2.81 × 10^3^ (*p* < 0.001); 95.4% reduction •Comparison between groups: no statistically significant difference (*p* > 0.05)
Zandi et al. [39]	2016	To compare the antibacterial effects of 1% NaOCl and 2% CHX	49 (29 males, 20 females, mean age = 50, age range 21–91)	49 (NaOCl: 20; CHX: 29)	Single-rooted and multi-rooted teeth (only one root for patient was considered)	Apical periodontitis (secondary treatment)	1 mm	Detectable bacteria: •1% NaOCl: 7/20 positive •2% CHX: 12/29 positive •No statistically significant difference between groups (*p* > 0.05) •Number of bacterial cells: •1% NaOCl: BT: 7.96 × 10^4^-AT: 2.95 × 10^2^ (*p* < 0.01)−99.6% reduction •2% CHX: BT: 5.37 × 10^5^-AT: 1.10 × 10^3^ (*p* < 0.01)−99.8% reduction
Ballal et al. [40]	2019	To assess whether dual rinse HEDP alter the clinical efficacy of NaOCl or adds any untoward clinical effects	60 (35 males, 25 females, age range 18–65 years)	60 (HEDP: 30; NaOCl alore: 30)	Single-rooted and multi-rooted teeth (only one root for patient was considered)	Asymptomatic apical periodontitis (primary treatment)	Determined using an electronic apex locator	Detectable bacteria •HEDP: BT: 30/30–AT: 15/30 •2.5% NaOC: BT: 30/30–AT: 12/30 (40%) •Comparison between groups after treatment: no statistically significant difference (*p* > 0.05)
Ballal et al. [41]	2020	To compare four NaOCl irrigation activation systems	80 (50 males, 30 females, mean age 41)	80 (PUI: 20; F-file: 20; XP-endo finisher: 20; Needle irrigation: 20)	Single-rooted and multi-rooted teeth (only one root for patient was considered)	Asymptomatic apical periodontitis with and without periapical lesions	Determined using radiographs and an apex locator	Cultivable Bacteria (CFUs/mL) •XP-endo Finisher: BT: median: 12.20; sd: 45.87–AT: median: 0.008; sd: 0.0001 •Needle irrigation: BT: median: 12.40; sd: 9.2–AT: median: 1.09, sd: 3.56 •F-files: BT: median: 20.65, sd: 69.23–AT: median: 0.34, sd: 4.72 •Ultrasonic: BT: median: 44.82, sd: 16.60–AT: median: 0.0055; sd: 0.032
Orozco et al. [42]	2020	To evaluate the effectiveness of passive ultrasonic irrigation compared to conventional needle irrigation	20 (10 females, 10 males)	20 (PUI: 10; Needle irrigation: 10)	Single-rooted and multi-rooted teeth (only one root for patient was considered)	Primary endodontic infection	1 mm	Cultivable Bacteria (CFUs/mL) •PUI: BT: 25.8 × 10^5^ ± 4.70 × 10^5^-AT: 42 ± 119 •Needle irrigation: BT: 2.31 × 10^5^ ± 4.70 × 10^5^-AT: 1.76 × 10^3^ ± 3.31 × 10^3^ •Comparison between groups after treatment: no statistically significant difference (*p* > 0.05)

*AT, After Treatment; BT, before treatment; PUI, Passive Ultrasonic Irrigation*.

Following the previous point, Figure 2 has been updated. To avoid repeating data “Outcome,” already reported in Table 2, the authors modified Figure 2, which appears corrected below.

**Figure 2:**
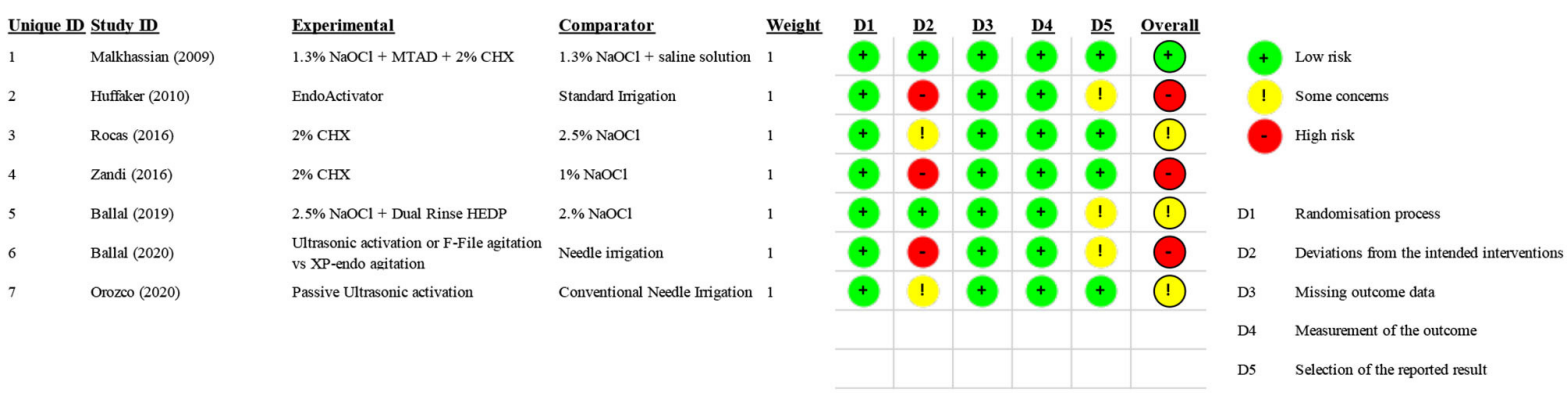
Risk of Bias—ROB2.

Following the previous points, the description in the original article has been updated. Two corrections have been made to section **Results**, subsection **Irrigating Solutions**. The corrected paragraphs appear below:

Rocas et al. [38] compared the effectiveness of 2% CHX with that of 2.5% NaOCl using a total volume of 15 mL for both irrigants but did not report the application time. In both groups, the mean number of bacterial cells decreased significantly after irrigation (*p* < 0.01). The rate of reduction in detectable bacteria was 40 and 44% in the treatment group (2% CHX) and in the control group (2.5% NaOCl), respectively. However, no statistically significant difference was observed upon comparing the mean number of bacterial cells between groups (*p* > 0.05) [38].

Zandi et al. [39] compared the effectiveness of 2% CHX with that of 1% NaOCl using a total volume of 10 mL for both irrigants but did not report the application time. In both groups, the mean number of bacterial cells decreased significantly after irrigation (*p* < 0.01), and the rate of reduction was higher than 99% (99.6% in the treatment group and 99.8% in the control group). However, no statistically significant difference was observed upon comparing the detectable bacteria between groups (*p* > 0.05).

The authors apologize for these errors and state that they do not change the scientific conclusions of the article in any way. The original article has been updated.

## Publisher's Note

All claims expressed in this article are solely those of the authors and do not necessarily represent those of their affiliated organizations, or those of the publisher, the editors and the reviewers. Any product that may be evaluated in this article, or claim that may be made by its manufacturer, is not guaranteed or endorsed by the publisher.

